# Astrovirus MLB2 Viremia in Febrile Child

**DOI:** 10.3201/eid1711.110496

**Published:** 2011-11

**Authors:** Lori R. Holtz, Kristine M. Wylie, Erica Sodergren, Yanfang Jiang, Carl J. Franz, George M. Weinstock, Gregory A. Storch, David Wang

**Affiliations:** Washington University School of Medicine, St. Louis, Missouri, USA

**Keywords:** astroviruses, viruses, viremia, febrile, child, diarrhea, enteric infections, plasma

## Abstract

Astroviruses cause diarrhea, but it is not known whether they circulate in human plasma. Astrovirus MLB2 was recently discovered in diarrhea samples from children. We detected MLB2 in the plasma of a febrile child, which suggests that MLB2 has broader tropism than expected and disease potential beyond the gastrointestinal tract.

Approximately 10% of nonbacterial, sporadic diarrhea is caused by infection with astroviruses (*1*). Until 2008, astroviruses that infect humans were thought to be limited to 8 closely related serotypes. However, 5 highly divergent astroviruses (MLB1, MLB2, VA1, VA2, and VA3) were discovered recently in stool samples from patients with (*2**–**4*) and without (*5*) diarrhea. No definitive disease association has been established for these 5 astroviruses.

A few reports have described enteric viruses in blood and other parts of the body, but none have described astroviruses in human plasma. For example, rotavirus RNA has been detected in serum (*6*), cerebrospinal fluid (*7*), and throat swab specimens (*7*); and rotavirus viral protein 6 and nonstructural protein 4 also have been detected in serum (*6**,**8*). Norovirus RNA also has been reported in human serum (*9*) and in cerebrospinal fluid (*10*), and enterovirus RNA has been reported in human serum (*11*). We report a case of astrovirus MLB2 viremia.

## The Study

As part of a broad effort to define the human virome, we performed high-throughput sequencing (Genome Analyzer IIX; Illumina Inc., San Diego, CA, USA) on several plasma samples from children with febrile illness (K.M. Wylie et al., unpub. data). The Human Research Protection Office, Washington University (St. Louis, MO, USA) approved this study. The case report in this article describes results generated from study of a 20-month-old boy with a history of transient and resolved neutropenia. The child was evaluated in the emergency department of St. Louis Children’s Hospital for petechial rash (3-day history), fever <40°C (1-day history), cough, and nasal congestion. He did not have vomiting or diarrhea.

The evaluation included a leukocyte count, with results (7.8 × 10^3^ cells/mm^3^) within the reference values and with a differential count of 26% bands, 59% neutrophils, 8% lymphocytes, 6% monocytes, and 1% atypical lymphocyte; blood culture results were negative. Nasopharyngeal swab specimen was negative for respiratory syncytial virus, influenza types A and B, parainfluenza, and adenovirus by fluorescent antibody testing, and culture results were negative for respiratory viruses. Chest radiograph was interpreted as showing mild peribronchial thickening, which may represent a viral process. In addition, plasma or blood samples from the patient were subjected to a battery of PCR screenings for the following viruses, the results of which were all negative: adenovirus; enteroviruses (Enterovirus ASR; Cepheid Inc., Sunnyvale, CA, USA); human herpesvirus 6 and 7; parvovirus B19 (RealStar Parvovirus B19 PCR Kit 1.0; Altona Diagnostics, Hamburg, Germany); human bocavirus; cytomegalovirus (whole blood); Epstein-Barr virus (whole blood); and JC, BK, WU, and KI polyomaviruses.

Total nucleic acid was extracted from 100 μL of the patient’s plasma by using the Roche (Indianapolis, IN, USA) MagNa Pure System and randomly amplified by using a sequence-independent PCR strategy as described (*12*). Amplicons were sheared and, following standard library construction, were sequenced by using the Genome Analyzer IIX (Illumina Inc.) according to the manufacturer’s protocol. Sequencing resulted in 6,394,424 sequence reads of 100 nt. When present in the sheared amplicons, the primer used for random amplification was removed, resulting in 83-nt sequences. From all reads, 238 had >80% nt identity to the partial MLB2 sequence in GenBank (GQ502192.1) when aligned by using Cross_match software (www.phrap.org/phredphrapconsed.html#block_phrap). In addition, 374 sequence reads with similarity to anelloviruses were detected in this sample by alignment of the reads to the GenBank NT and NR databases, using Cross_match and Blastx (http://blast.ncbi.nlm.nih.gov/Blast.cgi), respectively. Anelloviruses are commonly detected in human blood (*13*) and have no known disease association. No reads aligned with any other viruses, except endogenous human retrovirus sequences.

Given the number of sequence reads from the plasma sample that could be aligned with the 3,280-nt sequence of MLB2 (accession no. GQ502192.1) in GenBank, we reasoned that additional reads were likely to be present from parts of the MLB2 genome that had not yet been sequenced. To provide a complete reference genome for such an analysis, we sequenced the complete MLB2 genome from a previously described isolate (GenBank accession no. GQ502192.1) (*3*) from a stool sample by using a combination of reverse transcription PCR (RT-PCR), 3′ and 5′ rapid amplification of cDNA ends, and pyrosequencing on a genome sequencer (Roche) as described (*4*). The complete MLB2 genome of 6,119 nt, excluding the polyA tail, was confirmed by Sanger sequencing of overlapping RT-PCR amplicons and has been deposited in GenBank (accession no. JF742759).

Comparison of the high-throughput sequencing reads from the plasma to the complete genome yielded an additional 199 reads with >80% nt identity. Assembly of all reads yielded 10 contigs, with an average length of 305 bp, which aligned throughout the MLB2 genome (Figure). Conventional RT-PCR and quantitative TaqMan RT-PCR independently confirmed the presence of MLB2 in the plasma sample. The complete sequence of the capsid (open reading frame 2) of this plasma-derived MLB2 strain was obtained by RT-PCR (GenBank JF742760) by using primers designed from the stool-derived MLB2 strain. The capsid of the plasma-derived MLB2 strain has 99% nt identity with the stool-derived MLB2 strain. Of the 27 nt substitutions, 25 were synonymous. Because of limited quantities of the plasma sample, we were unable to sequence the complete genome of the plasma-derived MLB2.

**Figure:**
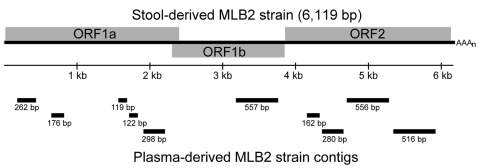
Map of 10 plasma-derived astrovirus MLB2 strain contigs generated by high-throughput sequencing (Genome Analyzer IIX; Illumina Inc., San Diego, CA, USA). ORF, open reading frame.

To quantify the MLB2 virus load in the plasma specimen, we developed a quantitative RT-PCR TaqMan assay targeting the capsid (forward primer LG0169 5′-ACAACTGGCCCTACATTGAATTC-3′, reverse primer LG0170 5′-CCGACACGCACATCTCGAT-3′, and probe FAM-TCGGGTCTTGGCGCGCGAT-tam). We used the MAXIscipt Kit (Ambion, Austin, TX, USA) to generate in vitro–transcribed RNA from a plasmid containing the region of interest to establish a standard curve for the assay. On the basis of the results of this assay, this sample has 4.5 × 10^5^ copies of MLB2 per mL of plasma.

To evaluate how frequently astroviruses may be present in human plasma, we screened archived plasma samples from 90 children with fever and 98 afebrile controls by using an astrovirus consensus RT-PCR (*3*). Total nucleic acid was extracted as described above. All 188 plasma samples were negative, which suggests that astrovirus MLB2 viremia is relatively rare, at least in the cohort analyzed.

## Conclusions

The role of novel astrovirus MLB2 in human health and disease and the clinical consequence of MLB2 viremia are not yet known. This case report raises the possibility that astrovirus MLB2 may be a cause of febrile illness. In addition, the finding of MLB2 viremia suggests that astrovirus MLB2 may have effects outside the enteric system. These data, combined with the recent detection of an astrovirus in brain tissue of an immunocompromised patient (*14*) and the brain tissue of mink with shaking mink syndrome (*15*), demonstrate a broader distribution of astroviruses in the body than previously recognized. The possibility that additional disease states may be linked to astrovirus infection is intriguing. For example, no other known pathogen was detected in the patient in this case report, and he had mostly upper respiratory signs, raising the possibility that MLB2 may play a role in respiratory illness. These new hypotheses warrant further investigation.
